# Can artificial intelligence-driven cephalometric analysis replace manual tracing? A systematic review and meta-analysis

**DOI:** 10.1093/ejo/cjae029

**Published:** 2024-06-19

**Authors:** Julie Hendrickx, Rellyca Sola Gracea, Michiel Vanheers, Nicolas Winderickx, Flavia Preda, Sohaib Shujaat, Reinhilde Jacobs

**Affiliations:** Department of Oral Health Sciences, Faculty of Medicine, KU Leuven, 3000 Leuven, Belgium; OMFS IMPATH Research Group, Department of Imaging and Pathology, Faculty of Medicine, KU Leuven, 3000 Leuven, Belgium; Department of Oral and Maxillofacial Surgery, University Hospitals Leuven, 3000 Leuven, Belgium; Department of Dentomaxillofacial Radiology, Faculty of Dentistry, Universitas Gadjah Mada, Yogyakarta 55281, Indonesia; Department of Oral Health Sciences, Faculty of Medicine, KU Leuven, 3000 Leuven, Belgium; Department of Oral Health Sciences, Faculty of Medicine, KU Leuven, 3000 Leuven, Belgium; OMFS IMPATH Research Group, Department of Imaging and Pathology, Faculty of Medicine, KU Leuven, 3000 Leuven, Belgium; Department of Oral and Maxillofacial Surgery, University Hospitals Leuven, 3000 Leuven, Belgium; OMFS IMPATH Research Group, Department of Imaging and Pathology, Faculty of Medicine, KU Leuven, 3000 Leuven, Belgium; Department of Oral and Maxillofacial Surgery, University Hospitals Leuven, 3000 Leuven, Belgium; King Abdullah International Medical Research Center, Department of Maxillofacial Surgery and Diagnostic Sciences, College of Dentistry, King Saud bin Abdulaziz University for Health Sciences, Ministry of National Guard Health Affairs, Riyadh 14611, Kingdom of Saudi Arabia; OMFS IMPATH Research Group, Department of Imaging and Pathology, Faculty of Medicine, KU Leuven, 3000 Leuven, Belgium; Department of Oral and Maxillofacial Surgery, University Hospitals Leuven, 3000 Leuven, Belgium; Department of Dental Medicine, Karolinska Institutet, 141 04 Stockholm, Sweden

**Keywords:** artificial intelligence, anatomic landmarks, cephalometry, orthodontics

## Abstract

**Objectives:**

This systematic review and meta-analysis aimed to investigate the accuracy and efficiency of artificial intelligence (AI)-driven automated landmark detection for cephalometric analysis on two-dimensional (2D) lateral cephalograms and three-dimensional (3D) cone-beam computed tomographic (CBCT) images.

**Search methods:**

An electronic search was conducted in the following databases: PubMed, Web of Science, Embase, and grey literature with search timeline extending up to January 2024.

**Selection criteria:**

Studies that employed AI for 2D or 3D cephalometric landmark detection were included.

**Data collection and analysis:**

The selection of studies, data extraction, and quality assessment of the included studies were performed independently by two reviewers. The risk of bias was assessed using the Quality Assessment of Diagnostic Accuracy Studies-2 tool. A meta-analysis was conducted to evaluate the accuracy of the 2D landmarks identification based on both mean radial error and standard error.

**Results:**

Following the removal of duplicates, title and abstract screening, and full-text reading, 34 publications were selected. Amongst these, 27 studies evaluated the accuracy of AI-driven automated landmarking on 2D lateral cephalograms, while 7 studies involved 3D-CBCT images. A meta-analysis, based on the success detection rate of landmark placement on 2D images, revealed that the error was below the clinically acceptable threshold of 2 mm (1.39 mm; 95% confidence interval: 0.85–1.92 mm). For 3D images, meta-analysis could not be conducted due to significant heterogeneity amongst the study designs. However, qualitative synthesis indicated that the mean error of landmark detection on 3D images ranged from 1.0 to 5.8 mm. Both automated 2D and 3D landmarking proved to be time-efficient, taking less than 1 min. Most studies exhibited a high risk of bias in data selection (*n* = 27) and reference standard (*n* = 29).

**Conclusion:**

The performance of AI-driven cephalometric landmark detection on both 2D cephalograms and 3D-CBCT images showed potential in terms of accuracy and time efficiency. However, the generalizability and robustness of these AI systems could benefit from further improvement.

**Registration:**

PROSPERO: CRD42022328800.

## Introduction

Cephalometric analysis provides important anatomical measurement data that is essential for orthodontic and craniomaxillofacial surgical workflows. It enables the morphometric quantification of craniofacial growth and the analysis of spatial relationships between hard and soft dentomaxillofacial structures for diagnostics, treatment planning, and outcome assessment [1, 2]. A standard cephalometric analysis is performed on two-dimensional (2D) lateral cephalograms or three-dimensional (3D) cone-beam computed tomography (CBCT) images [3]. Both 2D and 3D cephalometry analyses require manual localization of anatomical landmarks, which is a time-consuming task that can take approximately 15 min per case for an orthodontist [4]. Furthermore, the accuracy of landmark identification is subject to variability depending on the observer’s experience and image quality [5, 6].

Recently, solutions driven by artificial intelligence (AI), specifically machine learning (ML) and deep learning (DL), have been increasingly used to enhance the reliability, consistency, and accuracy of landmark placement for 2D and 3D cephalometric analyses [7, 8]. Machine learning, a subset of AI, creates algorithms that learn primarily from structured data, with decisions made based on intrinsic statistical patterns. Conversely, DL is a subset of ML that consists of convolutional neural networks (CNNs), a multilayer structure-learning algorithm that facilitates data processing through neural networks and automated data learning, akin to the functioning of human brain. In terms of performance, DL has demonstrated superiority over ML algorithms for various medical image analysis tasks. This is attributed to its capability to handle high-dimensional data of radiographic images with multiple predictor variables, and its ability to automatically and adaptively learn hierarchical features such as corners, shapes, and edges [9, 10].

As the identification of landmarks is one of the primary causes of error in cephalometric analysis owing to observer variability [6, 11], it is important to consider whether AI-driven solutions could serve as an accurate and time-efficient alternative to their traditional manual counterparts [12]. Despite numerous studies on automated landmarking for both 2D and 3D cephalometric analyses, we believe a gap exists in literature related to the comprehensive review of the accuracy of these AI-driven solutions. In this context, the accumulation of evidence could enhance our understanding of the accuracy of AI-driven solutions. Existing systematic reviews on this topic have either restricted their investigation to deep learning alone [8, 13], or exclusively focused on 3D images [13].

In the field of orthodontics, 2D landmarking and cephalometric analysis are often favoured due to their capacity to yield substantial data, which aids in devising the most effective treatment strategies for large portion of orthodontic patients. In these situations, 3D cephalometry derived from CBCT images is generally not advised, mainly because of the high radiation exposure risks [14, 15]. On the other hand, 3D cephalometry has advantages in terms of precise anatomical recognition and intricate structural assessment. This is particularly useful when more comprehensive treatment planning is required, such as in the digital planning processes of orthognathic surgery and implantology. In these cases, traditional 2D landmarking may not provide adequate information [16]. Hence, both types of datasets are considered clinically significant, depending on the specific task [17]. Despite the significant differences in AI methodologies and algorithms applied for automated 2D and 3D landmarking, a comprehensive review encompassing both types of datasets can offer an integrated view of the discipline. This approach could highlight progress in both dimensions and identify areas necessitating additional research and development.

Therefore, the aim of this systematic review and meta-analysis was to report the accuracy and efficiency of AI-driven automated landmark detection on 2D lateral cephalograms and 3D-CBCT images.

## Materials and methods

### Protocol and registration

The study protocol was registered under the number CRD42022328800 in the PROSPERO (Prospective Register of Systematic Reviews) database. The title and research question of the review were modified from their original version, as documented in PROSPERO (Supplementary File 1). However, the rest of the methodology remained unchanged. The systematic review and meta-analysis were conducted following the PRISMA (Preferred Reporting Items for Systematic reviews and Meta-Analyses) guidelines [18].

### Review question

The review question was formatted according to the PICO (Population, Intervention, Comparison, and Outcome) framework, as follows:

Patients (P): 2D lateral cephalograms or 3D-CBCT images of human subjects.

Intervention (I): AI-based algorithms for automated cephalometric landmarks identification.

Comparison (C): manual landmarking by experts (ground truth), where experts refer to either experienced dentists, clinicians, or orthodontists having expertise in cephalometric landmarking.

Outcome (O): success detection rate (SDR), mean radial error (MRE), computational time.

Review question: Does the AI-driven cephalometric analysis (I) on 2D cephalograms and 3D-CBCT images (P) offer improved accuracy and time-efficiency (O) compared to manual landmarking by an expert (C)?”

### Eligibility criteria

The review included all full-text diagnostic accuracy studies evaluating the performance of AI-driven algorithms for the automated detection of landmarks. The studies were selected based on the following inclusion criteria: (i) training and testing on 2D lateral cephalograms or 3D-CBCT images (with sufficient detail e.g. dataset size, image modality, AI algorithm, etc.) for automated detection of relevant landmarks, which are commonly applied for performing cephalometric analysis, such as nasion, orbitale, menton, pogonion, and subnasale. (ii) reporting of results as success detection rate (SDR) or mean radial error (MRE) in millimetres (mm) to determine clinical applicability. (iii) studies comparing automated with manual landmarking as a clinical reference. No restrictions were applied regarding the year and language of the publication.

Case reports, review papers, book chapters, letters, conference papers, and commentaries were excluded from the review. Additionally, studies that solely included landmarks that do not contribute to standard cephalometric analysis, such as craniometric points (asterion, pterion, ophistion, etc.), were not considered for this review.

### Information sources and search

An electronic search was performed in PubMed, Web of Science, and Embase up to the period of January 2024. A two-pronged search strategy was applied which consisted of combining the technique of interest (AI, ML, DL) and diagnostic target (landmark detection for cephalometric analysis). Each concept consisted of MeSH terms and keywords. The full search strategy is presented in Table 1.

**Table 1.** Search strategy on each database.

Database	Search strategy
*Concept #1 artificial intelligence*	
PubMed	‘Artificial Intelligence’[Mesh] OR ‘Artificial Intelligence’[tiab] OR ‘machine learning’[tiab] OR ‘deep learning’[tiab] OR ‘neural network*’[tiab] OR ‘automated’[tiab] OR ‘automatic’[tiab]
Web of Science	TS = (‘Artificial Intelligence’ OR ‘machine learning’ OR ‘deep learning’ OR ‘neural network*’ OR ‘automated’ OR ‘automatic’
Embase	‘artificial intelligence’/exp OR ‘artificial intelligence’:ti,ab,kw OR ‘machine learning’:ti,ab,kw OR ‘deep learning’:ti,ab,kw OR ‘neural network*’:ti,ab,kw OR ‘automated’:ti,ab,kw OR ‘automatic’:ti,ab,kw
Grey Literature	‘Artificial Intelligence’ OR ‘machine learning’ OR ‘deep learning’ OR ‘neural network*’ OR ‘automated’ OR ‘automatic’
*Concept #2 cephalometric analysis*	
PubMed	‘Cephalometry’[Mesh] OR ‘Cephalometry’[tiab] OR ‘craniometry’[tiab] OR ‘cephalometric*’[tiab] OR ‘landmark detection’[tiab]
Web of Science	TS = (‘Cephalometry’ OR ‘craniometry’ OR ‘cephalometric*’ OR ‘landmark detection’)
Embase	‘cephalometry’/exp OR ‘cephalometry’:ti,ab,kw OR ‘cephalometric*’:ti,ab,kw OR ‘craniometry’:ti,ab,kw OR ‘landmark detection’:ti,ab,kw
Grey Literature	‘Cephalometry’ OR ‘craniometry’ OR ‘cephalometric*’ OR ‘landmark detection’

A comprehensive grey literature search was executed using databases such as ProQuest, Google Scholar, OpenThesis, and OpenGrey to minimize the risk of selection bias. In addition, a thorough hand-search of references within original articles, reviews, and conference proceedings (collection of conference papers) was performed to identify any additional studies that were not retrieved from the chosen electronic databases. The articles identified were imported into Endnote X9 software (Thomson Reuters, Philadelphia, PA, USA) for the removal of duplicates and further selection.

### Study selection and data extraction

Two reviewers (J.H., M.V.) independently screened the relevant articles based on their titles and abstracts, followed by full-text reading of the included studies against the eligibility criteria. Any disagreement was resolved through discussion. A third experienced reviewer (R.J.) was consulted if consensus could not be reached.

Data extracted from the selected articles included: title, author, year of publication, country of origin, aim of the study (algorithm’s computational improvement or clinical validation), image type (2D lateral cephalograms or 3D-CBCT images), dataset source, total sample size, subsets (training, validation, test), characteristics of applied AI-based algorithm, number of landmarks and reported outcomes. The corresponding authors of the included studies were contacted for the provision of any further information or missing data.

## Risk of bias assessment

The Quality Assessment of Diagnostic Accuracy Studies-2 (QUADAS-2) tool was used to evaluate the risk of bias and applicability concerns. This tool was chosen due to its comprehensive coverage of aspects that need assessment in primary diagnostic accuracy studies, and its customizability, which allows for a more focused approach tailored to specific review. It served two purposes: first, to assess the impact of potential bias sources on test accuracy estimates, and second, to evaluate the influence of hypothesized sources of clinical heterogeneity on these estimates [19].

The tool consisted of a systematically developed checklist for determining the quality of diagnostic test accuracy studies (DTA). The checklist was divided into four domains for evaluating the risk of bias: (i) data selection (consecutive or random inclusions, no case-control design, no inappropriate exclusions); (ii) index test, i.e. test under evaluation (interpretation blinded for and independent of reference standard); (iii) reference standard, i.e. how was ground truth established (interpretation independent of and blinded for index test, valid reference test); (iv) flow and timing (sufficient time between index test and reference standard, did all data receive reference standard and the same one, all data included in the analysis). The first three domains were also evaluated in relation to concerns about applicability (does each domain match the research question) [19]. The applicability concerns help to determine if the study’s findings can be applied to real-life clinical scenarios. If significant concerns arise in any of the domains, it could impact the overall applicability of the study’s results to a broader patient population or clinical setting [20].

Two reviewers (J.H., M.V.) independently assessed the risk of bias using the QUADAS-2 checklist. Discrepancies were resolved through discussion. If consensus could not be reached, third experienced reviewer (R.J.) was consulted.

## Data analysis and synthesis

A meta-analysis was conducted using RStudio (version 2023.12.1, Posit Software, Boston, MA, USA) to evaluate the accuracy of 2D landmarks identification based on MRE and standard error (SE), where MRE value closer to zero corresponds to higher accuracy of automated landmarks identification. When multiple test datasets were used in the studies, they were assessed as separate groups to account for data variability. The summary measures included the MRE of test datasets with 95% confidence interval (CI). Heterogeneity was examined using *Q*-value and *I*^2^ statistics. The choice of statistical model was determined by the *I*² statistics, a measure of heterogeneity. If the *I*² was less than 50%, indicating low heterogeneity, a fixed-effects model was employed. Conversely, if the *I*² exceeded 50%, suggesting substantial heterogeneity, a random-effects model was utilized. The selected model was then used to generate the forest plot. The number of radiographs and cephalometric landmarks evaluated in each test dataset was considered when determining the weights of each study in the meta-analysis. A *P*-value of less than 0.05 was deemed statistically significant.

## Results

### Study selection

**Figure 1.**
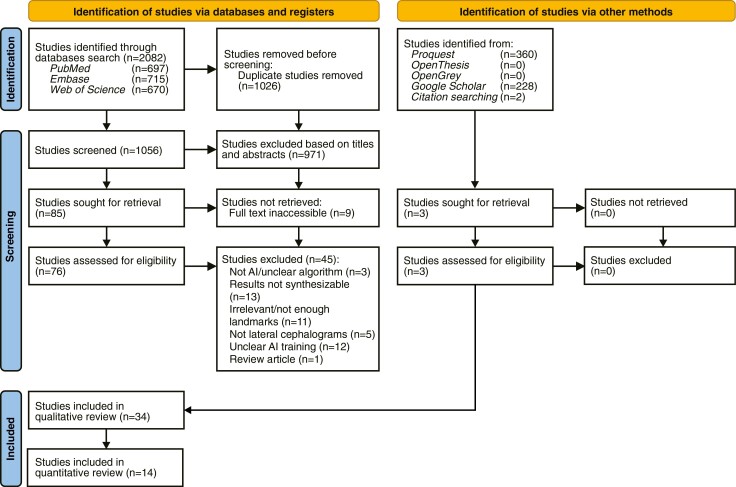
PRISMA 2020 flow diagram for systematic reviews.

The electronic database search yielded 2082 articles. Of these, 1026 were duplicates and 971 did not meet the eligibility criteria based on their titles and abstracts. The full text of the remaining 76 articles was reviewed, resulting in further exclusion of 45 articles. Supplementary File 2 describes the reasons for exclusion. Ultimately, 34 studies were deemed eligible and included in the systematic review. The selection process is depicted in the PRISMA 2020 flow diagram (Fig. 1).

### Study characteristics

The included studies covered a period of seven years, from 2017 until 2023. The majority of the studies originated from South Korea (*n* = 15), followed by China (*n* = 7), Japan (*n* = 3), USA (*n* = 3), Germany (*n* = 2), and one each from France, Hong Kong, Netherlands, and Turkey. Automated AI-based landmarks identification was applied on 2D lateral cephalograms in 27 studies and 3D-CBCT images in 7 studies. Most studies (27 studies) primarily investigated the computational improvement of algorithms for landmark detection, while seven studies focused on clinical validation of established methods. The characteristics of these 2D and 3D studies are summarized in Tables 2 and 3 respectively.

**Table 2.** Characteristics of included studies using 2D lateral cephalograms.

1st author	Year	Country of origin	Compu-tational improve-ment	Clinical validation	AI framework	Land-marks (N)	Data source	Total sample	Train set	Test set	Train and test set separation	Resolution (mm/pixel)	SDR within 2 mm range or MRE in mm	SDR of gonion within 2 mm range or MRE in mm	Computa-tional time
Arik *et al.* [27]	2017	USA	✓		Custom CNN combined with a shape model for refinement	19	IEEE Grand Challenge 2015	400	150	150 + 100	Yes	0.1	SDR Test 1: 75.37% SDR Test 2: 67.68%		
Bulatova *et al*. [51]	2021	USA		✓	CNN YOLOv3 by DDH Inc	16	AAOF Legacy Denver collection	110			n/a	n/a	SDR: 75%		
Chen *et al* [3]	2019	Hongkong	✓		VGG-19, ResNet20, and Inception; custom attentive feature pyramid fusion and regression voting	19	IEEE Grand Challenge 2015	400	150	150 + 100	Yes	0.1	Test 1: SDR: 86.67% MRE: 1.17 ± 1.19 mm Test 2: SDR: 75.05% MRE: 1.48 ± 0.77 mm		−
Chen *et al* [52].	2023	China	✓		U-shpaed CNN with Monte Carlo dropout	19	IEEE Grand Challenge 2015 + own dataset (108)	508	258	150 + 100	Yes	0.1	Test 1: SDR: 79.65% MRE: 1.39 ± 1.06 mm Test 2: SDR: 80.05% MRE: 1.33 ± 0.93 mm	Test 1: SDR: 55.33% MRE: 2.15 ± 1.50 mm Test 2: SDR: 52.00% MRE: 2.20 ± 1.36mm	0.75 s
Hong *et al* [53].	2023	Korea	✓		Deep Q-network	19, 36	IEEE Grand Challenge 2015 + Samsung Medical Center (SMC) dataset (500)	900	150 + 420	150 + 100 + 80	Yes	0.1, 0.13	IEEE: Test 1: SDR: 77.65% MRE: 1.49 ± 1.42 mm Test 2: SDR: 70.47% MRE: 1.60 ± 1.3 mm SMC dataset: SDR:67.33% MRE: 1.89 ± 1.5 mm		
Huang *et al*. [54]	2021	Germany	✓		LeNet-5 for ROI patches and ResNet50 for landmark location	19	CQ500 CTs (train) and IEEE Grand Challenge 2015 (test)	741	491	150 + 100	Yes	0.5	Test 1: SDR: 86.7% Test 2: SDR: 73.7%		
Hwang *et al*. [25]	2021	Korea		✓	Customized YOLO V3	19	n/a	2183	1983	200	Yes	n/a	SDR: 75.45% MRE: 1.76 ± 2.16 mm	SDR: 38.0% MRE: 2.75 ± 1.84 mm	
Kim *et al* .[42]	2020	Korea	✓		Stacked houglass-shaped DL model	23	Own dataset, IEEE Grand Challenge 2015	2475	Training: 1675 Validation: 175 Fine-tuning: 300 (IDBI)	225 + 100 (ISBI)	Yes	Own dataset: 0.127-0.15 ISBI: 0.1	SDR: 84.53% Group 1 (test set: 200): MRE: 1.37 ± 1.79 mm		0.4s
Kim *et al.* [55]	2021	Korea	✓		Multistage CNN	15	Kyung Hee University Dental Hospital	860	690	170	Yes	0.39	SDR: 87.13% MRE: 1.03 ± 1.288 mm	SDR: 62.64% MRE: 2.04 ± 1.727 mm	
Kim *et al*. [56]	2021	Korea		✓	DL model with a 2-step structure: a ROI machine and a detection machine	13	Yonsei Dental Hospital	950	Training: 800 Validation: 100	50	Yes	0.12	SDR: 64.3% MRE: 1.84 mm		−
Kwon *et al* . [32]	2021	Korea	✓		Multistage probabilistic approach based on DeepLabv3	19	IEEE Grand Challenge 2015	400	Training:150 Validation: 150)	100	Yes	0.1	Validation test: SDR: 86.91% MRE: 1.12 mm Test 2: SDR: 77.16% MRE: 1.41 mm	Test 2: SDR: 85.00% MRE: 1.20 mm	
Le *et al.* [39]	2022	Korea		✓	Deep Anatomical Context Feature Learning (DACFL)	41	Jeonbuk National University Dental Hospital	1293	1193	100	Yes	n/a	SDR: 73.32% MRE: 1.87 ± 2.04 mm	SDR: 51% MRE: 2.70 ± 2.14 mm	
Lee *et al*. [7]	2020	Korea	✓		Custom CNN for ROI and custom Bayesian CNN for landmark detection	19	IEEE Grand Challenge 2015	400	150	250	Yes	0.1	SDR: 82.11% MLE (landmark): 1.53 ± 1.74 mm	SDR: 63.33% MLE: 2.39 ± 4.77 mm	512s (with 1 GPU) 38s (with 4 GPUs)
Lee et al [57]	2022	Korea	✓		Single-passing CNN for an accurate regression of the landmarks	19	IEEE Grand Challenge 2015	400	150	150 + 100	Yes	0.1	Test 1: SDR: 86.42% MRE: 1.19 ± 0.80 mm Test 2: SDR: 74.58%	Test 1: SDR: 60.67% Test 2: SDR: 77.00%	
Noothout *et al*. [58]	2020	Netherlands	✓		Custom FCNNs based on ResNet34	19	IEEE Grand Challenge 2015	400	Training: 140 Validation: 10	150 + 100	Yes	0.1	Test 1: SDR: 82% Test 2: SDR: 72%	Test 1: MRE: 2.12 ± 1.83 mm Test 2: MRE: 1.68 ± 1.61 mm	0.05 ± 0.009 s
Oh *et al*. [59]	2021	Korea	✓		DACFL, custom FCN combined with a local feature perturbator with anatomical configuration loss	19	IEEE Grand Challenge 2015	400	150	150 + 100	Yes	0.1	Test 1: SDR: 86.20% Test 2: SDR: 75.89%	Test 1: SDR: 60.1% Test 2: SDR: 83.0%	0.15s
Park *et al*. [60]	2019	Korea		✓	YOLO V3 with modification and single shot multibox detector (SSD)	80	Seoul National University Dental Hospital	1311	1028	283	Yes	0.14	YOLOv3: SDR: 80.4%	−	YOLOv3: 0.05s SSD: 2.89s
Qian *et al*. [28]	2020	China	✓		Cepha-NN, combining U-Net-shaped networks, attention mechanism, and region enhancing loss	19	IEEE Grand Challenge 2015	400	150	150 + 100	Yes	0.1	Test 1: SDR: 87.61% MRE: 1.15 mm Test 2: SDR: 76.32% MRE: 1.43 mm	Test 1: SDR: 67.33% MRE: 1.5941 mm Test 2: SDR: 81.00% MRE: 1.3809 mm	
Song *et al*. [29]	2020	Japan	✓		Two-step approach: ROI extraction and ResNet50	19	IEEE Grand Challenge 2015, Sandong University for testing	500	150	150 + 100 + 100 (own dataset)	Yes	0.1	Test 1: SDR: 86.4% MRE: 1.077 mm Test 2: SDR: 74.0% MRE: 1.542 mm Own dataset: SDR: 62.0% MRE: 2.1 mm	Test 1: SDR: 62.7% MRE: 1.817 mm Test 2: SDR: 75.0% MRE: 1.431 mm Own dataset: SDR: 51.0% MRE: 2.4 mm	
Song *et al*. [61]	2021	Japan	✓		U-Net based with encoders and decoders and a second fine detection step	19	IEEE Grand Challenge 2015	400	150	150 + 100	Yes	0.1	Test 1: SDR: 85.2% MRE: 1.194 mm Test 2: SDR: 72.2% MRE: 1.643 mm	Test 1: SDR: 60.0% MRE: 1.966 mm Test 2: SDR: 67.0% MRE: 1.999 mm	4.0s
Ugurlu *et al*. [62]	2022	Turkey	✓		CranioCatch: feature aggregation and refinement network (FARNet), CNN based DL model	21	Departement of Ortho-dontics, Faculty of Dentistry, Eskişehir Osmangazi University	1620	Training: 1300 Validation: 140	180	Yes	n/a	SDR: 76.2% MRE: 3.400 ± 1.57 mm	SDR: 48.3% MRE: 8.304 ± 2.98 mm	
Wang *et al*. [63]	2021	China	✓		DCNN based on a iterative method	19	IEEE Grand Challenge 2015	300	150	150	Yes	0.1	SDR: 87.51%	SDR: 74.7%	20s
Yang *et al* [2].	2023	Korea	✓		CephNet with FCN	19	Seoul National University	1286	Training: 704 Validation: 182	400	Yes	0.1	SDR: 73.14% MRE: 1.75 ± 1.67 mm	SDR: 74.00% MRE: 2.13 ± 3.47 mm	
Yao *et al*. [30]	2022	China	✓		CNN with a global detection module and a locally modified module	37	Department of oral and maxillofacial surgery, West China College of stomatology Sichuan University	512	Training: 312 validation: 100	100	Yes	0.125	Validation data: SDR: 97.30% MRE: 1.127 ± 1.02 8 mm Test: SDR: 97.30% MRE: 1.038 ± 0.893 mm	Test set: SDR: 65% MRE: 1.721 ± 1.325 mm	3s
Zeng *et al*. [64]	2021	China	✓		Cascaded three-stage CNN	19	IEEE Grand Challenge 2015 + own dataset (102)	502	150	150 + 100 + 102	Yes	0.1	Test 1: SDR: 81.37% MRE: 1.34 ± 0.92 mm Test 2: SDR: 70.58% MRE: 1.64 ± 0.91 mm Extra validation: SDR: 64.81% MRE: 2.02 ± 1.89 mm	Test 1: SDR: 57.33% MRE: 1.97 ± 1.10 mm Test 2: SDR: 69.00% MRE: 1.59 ± 1.02 mm	
Zhao *et al* [65].	2023	China	✓		Multi-scale YOLO V3	19	IEEE Grand Challenge 2015	400 Augmented to 2100	1950	150	Yes	0.1	SDR: 80.84%	SDR: 55.33%	
Zhong *et al*. [66]	2019	China	✓		2-stage (global and local) U-Net models	19	IEEE Grand Challenge 2015	400	150	150 + 100	Yes	0.1	Test 1: SDR: 86.91% MRE: 1.12 ± 0.88 mm Test 2: SDR: 76.00% MRE: 1.42 ± 0.84 mm		

Abbreviations: MRE, mean radial error; SDR, success detection rate.

**Table 3.** Characteristics of included studies using 3D-CBCT images.

1st author	Year	Country of origin	Computa-tional improve-ment	Clinical validation	AI framework	Land-marks (N)	Data source	Total sample	Train set	Test set	Train and test set separation	Resolution (mm^3^/voxel)	SDR within 2 mm range or MRE in mm	SDR of gonion within 2 mm range or MRE in mm	Computa-tional time
Dot *et al*. [67]	2022	France	✓		DL method based on Spatial Configuration-Net (SCN)	33	Own dataset	198	Training: 128 Validation: 32	38	Yes	0.45	SDR: 90.4% MRE: 1.0 ± 1.3 mm	SDR: L: 70.3% R: 48.7% MRE L: 1.9 ± 1.7 mm R: 2.1 ± 1.4 mm	60s
Lang *et al.* [23]	2022	USA	✓		DL method extending Mask R-CNN	105	n/a	50	−	−	n/a	0.4	MSE (squared): 1.38 ± 0.95 mm	−	−
Lee *et al*. [22]	2019	Korea	✓		VGG-19 (DL)	7	Own dataset	27	20	7	Yes	n/a	Avarage point-to-point error: 1.5 mm	−	−
Ma *et al*. [31]	2020	Japan		✓	Patch-based deep neural networks with a three-layer CNN	13	The University of Tokyo Hospital	66	58	8	Yes	0.35	Avarage landmarking error: 5.785 ± 0.980 mm	Landmarking error L: 5 mm R: 4 mm	37.871 ± 3.766s
Yun *et al*. [26]	2020	Korea	✓		Custom CNNs, combined skull normalization, with variational autoencoder (VAE) for coarse to fine detection tasks	93	Own datasets	26 + 229	22 + 208	4 + 21	Yes	n/a	3D point-to-point error: 3.63 mm	−	−
Yun *et al*. [24]	2022	Korea	✓		Semi-supervised DL method	90	Yonsei University	24	15	9	Yes	n/a	MDE (detection): 2.88 mm	−	−
Weingart *et al* .[68]	2023	Germany		✓	Deep Neural Patchwork	60	University Hospital Freiburg	30	15	15	Yes	n/a	SDR: 66.4% Mean error: 1.94 ± 1.45 mm	−	2 min

Abbreviations: MRE, mean radial error; SDR, success detection rate.

Almost half of the 2D studies evaluated the accuracy of their AI algorithms using a public benchmark dataset from the IEEE International Symposium on Biomedical Imaging 2015 grand challenge [21]. This dataset consisted of 400 high-resolution lateral cephalograms (training set = 150, test set 1 = 150, test set 2 = 100) with 19 manually annotated landmarks by two experts (1 junior and 1 senior orthodontic specialist) as the ground truth. These manually annotated landmarks serve as a reference against which the AI algorithm’s performance is measured.

**Figure 2.**
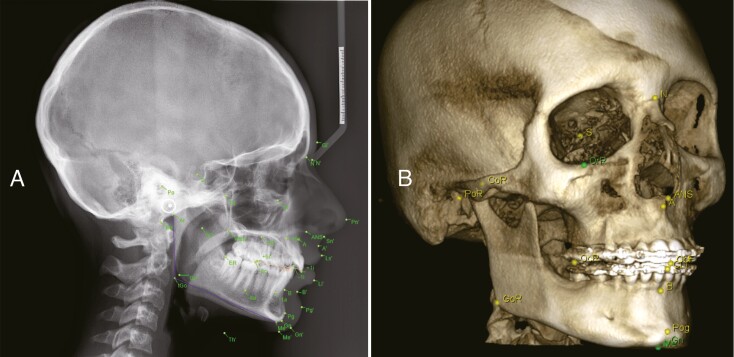
Cephalometric landmarks detection with Romexis module showing common landmarks for performing cephalometric analysis, such as nasion, orbitale, menton, pogonion, gnathion, point A, point B, anterior nasal spine, porion, sella, and gonion; (A) automated two-dimensional landmarking, (B) manual three-dimensional landmarking.

The original dimensions of the images were 1935 × 2400 pixels, with resolution of 0.1 mm per pixel in both horizontal and vertical directions. The average intra-observer variability for these landmark points was found to be 1.73 mm for the junior expert and 0.90 mm for the senior expert. On the other hand, the inter-observer variability between both experts was found to be 1.38 mm, suggesting reasonable accuracy target for automated landmark detection techniques. To compensate for any inter-observer variability, the mean position of the two points from both experts was used as the ground truth [21]. Among the included studies, the total number of landmarks tested ranged from 7 [22] to 105 [23]. The amount of data for training ranged from 15 [24] to 1983 images [25], while the test dataset ranged from 4 [26] to 400 images [2]. Figure 2 illustrates an AI-derived automated landmark identification on 2D cephalogram followed by manual correction by an expert, and manual identification on 3D-CBCT image.

### Qualitative synthesis

A qualitative synthesis of all reported data related to automated 2D and 3D landmarks identification was conducted. The 2D studies, which used only the IEEE dataset, demonstrated that the accuracy of test set 1 ranged from 75.37% [27] to 87.61% [28], based on the SDR value within the 2 mm error threshold. Conversely, 18 studies that used their own datasets, either alone or in combination with the IEEE dataset, revealed SDR ranging from 62.0% [11, 29] to 97.30% [30] within the clinically acceptable 2mm range.

Studies that applied automated landmarking on 3D-CBCT images reported their accuracy as either mean error (*n* = 7) or SDR (*n* = 2), with the highest observed error being 5.785 mm [31]. Of all the landmarks on 2D and 3D images, gonion was generally the most challenging to locate automatically, where the lowest SDR was 38.0% [25], and the highest was 85.00% [32] within the 2 mm threshold. The computational time to automatically detect the landmarks was calculated in 11 articles, all of which reported a timing of less than 1 min.

Table 4 presents list of cephalometric analysis that could potentially be performed using the automated landmarking proposed in the included studies. In terms of clinical applicability, the AI algorithms for automated landmark identification used in most studies could facilitate at least the Steiner and Down analyses. This was due to the algorithms’ ability to identify the following landmarks: sella, nasion, point A, point B, pogonion, gnathion, menton, gonion, porion, orbitale, upper incisor, and lower incisor [33, 34].

**Table 4.** Potential cephalometric analysis using annotated landmarks.

Cephalometric Analysis	References
Steiner	Chen *et al*. [3], Chen *et al*. [52], Hong *et al*. [53], Lee *et al*. [7], Lang *et al*. [23], Yun *et al* [24], Hwang *et al* [25], Yun *et al* [26], Park *et al* [60], Arik *et al* [27], Qian *et al* [28], Yao *et al* [30], Kwon *et al* [32], Lee *et al* [57], Oh *et al* [59], Huang *et al* [54], Kim *et al* [42], Kim *et al* [56], Le *et al* [39], Noothout *et al* [58], Song *et al* [29], Song *et al* [61], Ugurlu *et al* [62], Wang *et al* [63], Yang *et al*. [2], Zeng *et al* [64], Zhong *et al* [66], Zhao *et al*. [65], Dot *et al* [67], Weingart *et al* [68]
Down	Chen *et al* [3], Chen *et al*. [52], Hong *et al*. [53], Lee *et al* [7], Lee *et al* [22], Lang *et al* [23], Yun *et al* [24], Hwang *et al* [25], Yun *et al* [26], Park *et al* [60], Arik *et al* [27], Qian *et al* [28], Yao *et al* [30], Lee *et al* [57], Oh *et al* [59], Huang *et al* [54], Kim *et al* [42], Kim *et al* [55], Kim *et al* [56], Le *et al* [39], Noothout *et al* [58], Song *et al* [29], Song *et al* [61], Ugurlu *et al* [62], Wang *et al* [63], Yang *et al*. [2], Zeng *et al* [64], Zhao *et al*. [65], Zhong *et al* [66], Dot *et al* [67], Weingart *et al* [68]
Wits appraisal	Hong *et al*. [53], Lang *et al* [23], Yun *et al* [24], Yun *et al* [26], Park *et al* [60], Yao *et al* [30], Le *et al* [39], Dot *et al* [67], Weingart *et al* [68]
Tweed	Hong *et al*. [53], Lang *et al* [23], Yun *et al* [24], Yun *et al* [26], Park *et al* [60], Yao *et al* [30], Kim *et al* [55], Le *et al* [39], Dot *et al* [67], Weingart *et al* [68]
Ballard	Hong *et al*. [53], Yun *et al* [24], Yun *et al* [26], Park *et al* [60], Yao *et al* [30], Bulatova *et al* [51], Le *et al* [39], Dot *et al* [67], Weingart *et al* [68]

### Quantitative synthesis

**Figure 3.**
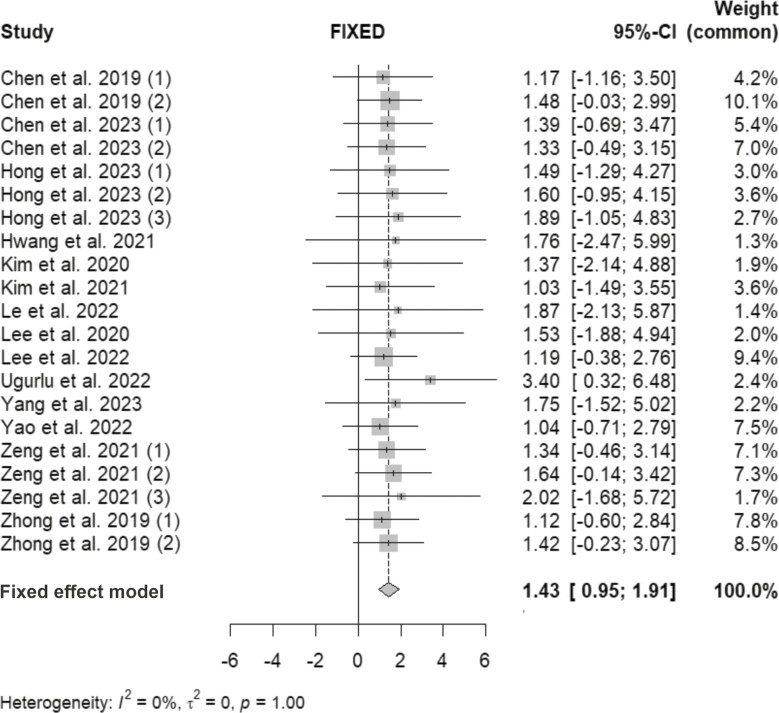
Forest plot of automated landmark identification on 2D cephalograms, reporting their accuracy in mean radial error (MRE) and standard error (SE) (mm). Studies using multiple test datasets are also indicated accordingly. Horizontal line indicates 95% confidence interval (CI), square shape indicates SE and diamond shape indicates pooled subtotal.

The meta-analysis was limited to the accuracy of 2D landmarks identification due to the diverse range of study designs and reported outcomes used in 3D cephalometry. The accuracy of AI-based 2D landmarks identification was evaluated in studies that reported the MRE and SE outcomes of test datasets. A total of 14 studies with 21 estimates were included, in which 3 studies tested their accuracy on 2 test sets and 2 studies on 3 sets. The statistical analysis revealed homogeneity among the included studies, as indicated by *Q*-value of 2.53 (*P* > 0.05) and *I*^2^ of 0%, indicating no significant heterogeneity among the studies. A fixed-effects model was employed since the included studies demonstrated homogeneity with the same true effect size. The results indicated that the prediction of AI-based landmark placement generally fell below the 2 mm error threshold (1.43; 95% CI: 0.95–1.91), and only the results of two studies exceeded this threshold (Fig. 3).

### Risk of bias assessment

**Figure 4.**
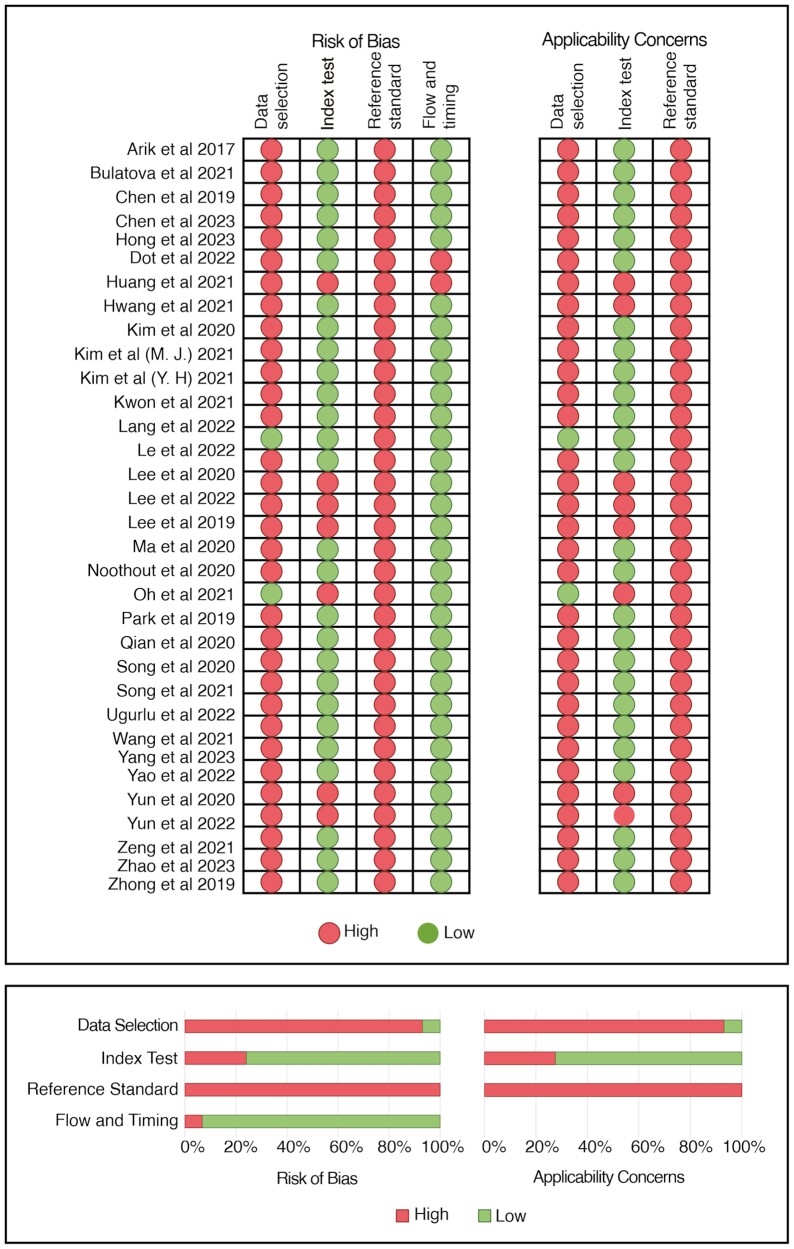
Risk of bias and applicability concerns based on Quality Assessment of Diagnostic Accuracy Studies-2 tool.

When using the QUADAS-2 tool, ‘AI-driven cephalometric landmark detection’ acted as the index test domain and ‘manual landmark placement by experts’ was considered as the reference standard domain. Most studies had a high risk of bias associated with data selection (93%), primarily because the authors did not employ randomized selection process. Furthermore, high risk also existed based on the use of the reference standard. Generally, the applicability concern associated with the included studies was high, with the exception of the index test usage. Fig. 4 provides a comprehensive overview of the risk of bias and applicability concerns.

## Discussion

In the digital age and the rise of precision dentistry, workflows in dentomaxillofacial practices are increasingly streamlined through the incorporation of AI-based technologies. This systematic review and meta-analysis were conducted to evaluate the accuracy of AI-powered tools in automating 2D and 3D cephalometric landmarking. A significant portion of the studies included in the review originated from East Asia (76.5%), with less representation from Europe and America. This trend can be attributed to various factors, such as the rapid advancement of technology and significant investment in AI research in East Asia, as emphasized in reports by the Organisation for Economic Co-operation and Development (OECD) and the Government AI Readiness Index. The position of East Asia as a leading global centre for AI innovation is evident from its extensive production of AI-related publications and its high ranking in the government AI readiness index These factors highlight East Asia’s crucial role in propelling AI research and development [35, 36]. Nevertheless, it is crucial to ensure broad spectrum of viewpoints and contributions in AI research, as this can result in more holistic and inclusive solutions. Therefore, there is a call for international collaborative research to ensure the universal applicability of AI technologies across varied patient demographics.

The findings of the review suggested variability in the accuracy of landmark detection amongst different studies. This could be attributed to the differences in the sample size used in the training set, where large heterogeneous samples with anatomical variabilities are expected to provide a more comprehensive learning process, thereby ensuring accuracy [37]. Moreover, each study used a distinct dataset for testing, separate from the one used for training. This is a normal practice in the evaluation of AI models. It ensures that the models are tested on data not encountered during the training process, thus minimizing the chance of overfitting and providing solid assessment of their ability to generalize [38]. However, it is important to note that these studies did not provide detailed descriptions of the specific methods employed to select the subjects included in the test dataset.

Most 2D cephalometric studies used the publicly accessible IEEE dataset to train AI algorithms, with the aim of enhancing the accuracy and efficiency of automatic landmark identification through computational improvements. Although the IEEE dataset offers the advantage of standardized performance comparability, it also introduces a challenge due to limited generalizability. Hence, making the clinical applicability of the AI tool questionable [39]. This issue was corroborated by studies included in the review that emphasised on clinical validation. These studies used their own datasets and demonstrated lower accuracy compared to those that focused on computational enhancement using the IEEE dataset [29]. Therefore, future research should utilize multi-centre datasets with varying acquisition parameters for clinical validation. This approach could enhance the consistency and robustness of AI-driven solutions and address generalisability issues, which are crucial for clinical applicability [40].

In the field of AI, particularly in the context of medical imaging and analysis, the complexity of a dataset is also determined by several other factors, such as size and shape of the anatomical structures, age, gender, type of malocclusion, ethnic background, and bone density [41, 42]. These characteristics introduce wide range of variations that the AI system must be able to recognize and interpret correctly [43]. This requires sophisticated algorithms and large amount of diverse training data. The more complex the dataset, the more challenging it can be for the AI to learn and make accurate predictions [37, 38, 44]. For instance, Tanikawa *et al*. [44] demonstrated that the performance of AI-driven automated landmarking was lower in patients with cleft palate compared to those without this condition. When training AI algorithms for cephalometric landmark detection, it is crucial to understand that the robustness and accuracy of the algorithm depend on its adaptability to variations encountered in clinical practice.

When comparing the included studies, a negative correlation was found between the reported accuracy and the size of training data. For instance, Hwang *et al*. trained the AI algorithm with 1983 2D images, each containing 19 annotated landmarks, and observed an accuracy of 73.2% [25]. In contrast, Lee *et al*. used a training set of 150 images and achieved higher accuracy of 86% [22]. This inconsistency could be associated with the variability of the training and testing sets, where Lee *et al*. relied on the IEEE dataset consisting of patients without any craniofacial deformities and with similar radiological patterns in both training and testing sets [43] On the other hand, Hwang *et al*.‘s testing datasets included patients with variable heterogeneous radiological patterns, which the AI algorithm might not have accurately identified based on the homogeneous dataset used for training.

Most 2D studies reported an accuracy of more than 80% within 2 mm error threshold, while the mean error of 3D landmark detection approximately ranged between 1. 0mm and 5.8mm. It is important to note that the accuracy of landmarking cannot be directly compared between these two types of datasets. In 2D imaging, landmarks are projected onto a single plane, simplifying the identification process and often leading to higher reported accuracy rates within the given error threshold. Conversely, 3D landmark detection involves identifying points within a volumetric space, which introduces additional complexity and challenges [24, 45]. Despite achieving high accuracy level, the performance of AI has not yet reached the level of an expert, and further improvements are anticipated, especially in the realm of 3D landmark detection. This is an area where limited number of cases were used for the training and testing of AI algorithms in the reviewed studies. Given the challenge of accurately detecting landmarks in three dimensions using small datasets, while still maintaining high accuracy within 2 mm error threshold, it is advisable to conduct additional studies with a larger sample size [31].

The findings of the included studies were compared against a threshold of 2 mm, which is generally accepted as clinically acceptable for most cephalometric measurements [6]. This tolerance for error is justified due to the inherent limitations of 2D imaging, which involves projected image of the majority of cephalometric points in the context of right-left asymmetry. Mostly, clinicians estimate a median between the projections of paired cephalometric points to establish the references for the cephalometric analysis. Although 3D imaging avoids geometrical distortion, precise segmentation from CBCT has not yet to be standardised in semiautomatic workflows [46, 47]. A discrepancy of even 2 mm can indeed have significant implications, especially when dealing with smaller patient sizes or specific landmarks. This is why it is crucial to strive for the highest accuracy possible in these situations. It is worth noting that while certain level of error might be deemed acceptable by clinical standards, the goal should always be to minimize this as much as possible to ensure the best patient outcomes. Moreover, clinicians are cognizant of potential errors in the placement of landmarks, which are typically taken into account subjectively during the clinical interpretation of the analysis and patient’s treatment planning [6].

The selection of the cephalometric landmarks included in our review was primarily based on their widespread use in orthodontics and clinical relevance [32, 33]. Among the different annotated landmarks on both 2D and 3D images, gonion was generally one of the most difficult landmarks to localise automatically and had the lowest detection rate. The identification of gonion appears to pose a significant challenge not only for AI algorithms, but also for human observers. This is primarily due to the fact that this landmark is a constructed point on the 2D cephalogram, resulting from the imperfect overlay of the bilateral aspects of the mandible. Additionally, the 3D error may be a consequence of discrepancies in volumetric segmentation or difficulties in determining the definitive vertical position of gonion along broadly curved structures, a problem also commonly encountered by human observers [48]. Hence, it is important to take these limitations into consideration while training an AI algorithm as such to improve its performance. It is worth noting that the accuracy of landmarks identification is heavily dependent on the expertise and anatomical knowledge of the experts [6]. Consequently, the experts responsible for creating training datasets should have substantial experience in this field. A low detection rate of the gonion point might diminish the overall measured performance of the AI tool. Hence, it is essential to address this issue to ensure the effectiveness of the AI tool.

Regarding clinical applicability, the AI algorithms discussed in most studies have demonstrated the ability to identify key landmarks commonly used in two of the most prevalent cephalometric analyses, namely Steiner and Down’s analyses [33, 34]. This suggests that the current AI tools could be considered clinically applicable for cases requiring orthodontic diagnosis and treatment planning. However, caution is advised as their accuracy has not yet reached the level of an expert, which could lead to errors in cephalometric analysis for diagnostics, planning, or outcome evaluation. Furthermore, time consumption is another important parameter to be considered in a clinical practice. While it takes an expert approximately 20 min to manually identify cephalometric landmarks [37], most AI-based algorithms can do so in less than a minute. Despite this, further research is needed to enhance AI’s accuracy, as time efficiency alone is not sufficient justification until it can provide accuracy comparable to that of an expert. A beneficial addition to AI algorithms would be the ability to identify which landmarks increase time consumption and are incorrectly identified. This would allow for manual intervention to correct these errors and train the algorithm based on the corrected data [39]. Incorporation of this human-AI collaboration for error correction should be considered in future studies.

This review encountered several limitations. First, the number of studies included was relatively limited, particularly those related to 3D landmarking. Second, due to the variability in the datasets, imaging parameters, and algorithms, the results of the quantitative synthesis should be interpreted with caution. Third, a significant risk of bias was observed in patient selection. While few studies provided detailed information about patient selection, the majority relied on IEEE dataset without explicitly outlining their sampling procedures [16, 38]. Finally, the manual identification of landmarks for the training set is subject to both inter- and intra-observer variability [49]. Hence, it is advisable to specify the training and calibration protocol for landmark identification when creating the ground truth for training. Future studies should also adhere to AI standards such as CONSORT-AI and SPIRIT-AI [50].

## Conclusions

The AI-driven cephalometric landmark detection on 2D and 3D images exhibited high accuracy and time efficiency. Although the majority of 2D studies indicated superior automated landmark detection performance, the error rates displayed by 3D studies were inconsistent, thus implying a need for further improvement. Moreover, clinicians are advised to remain vigilant due to the risk of inaccurate landmarks identification. To enhance the generalisability and clinical applicability of AI models, it is suggested that datasets be broadened to include a more diverse range of data. The incorporation of AI-driven landmarks identification in further studies could accelerate its refinement and overall development, thereby setting the stage for its potential to replace manual landmarking.

## Supplementary Material

cjae029_AQ16_Supplementary_File_1

cjae029_AQ16_Supplementary_File_2

## Data Availability

The data supporting this article will be shared upon reasonable request to the corresponding author.
