# Supplementary material for: Can artificial intelligence-driven cephalometric analysis replace manual tracing? A systematic review and meta-analysis
Source: Eur J Orthod. 2024 Jun 19;46(4):cjae029. doi: 10.1093/ejo/cjae029 (PMC11185929; doi:10.1093/ejo/cjae029)
Supplement: cjae029_AQ16_Supplementary_File_1 [file cjae029_aq16_supplementary_file_1.docx]

**Supplementary File 1**

Deviation from previous protocol:

1. Title

The previous title, “The use of artificial intelligence in 2D and 3D cephalometric analysis,” implied a broad exploration of AI applications in cephalometric analysis. In contrast, the new title, “Can AI-driven cephalometric analysis replace manual tracing? A systematic review and meta-analysis,” specifies a more focused research question. This shift in focus is crucial for clearly communicating the study’s objectives and scope to readers.

1. Review question

The review question has been refined to align more closely with the specific aims of the study, providing a clear framework for evaluating the effectiveness of AI-driven cephalometric analysis compared to manual landmarking by experts. By structuring the question according to the PICO framework, the study aims to investigate the accuracy and time-efficiency of AI-driven cephalometric analysis on 2D cephalograms and 3D-CBCT images compared to manual landmarking by experts. This structured approach enhances the clarity and focus of the research question, allowing for a more targeted analysis of the outcomes related to success detection rate, mean radial error, and computational time.

1. Search
   The revised search strategy extends the search period up to January 2024 and includes additional databases such as ProQuest, Google Scholar, OpenThesis, and OpenGrey to ensure comprehensive coverage of the literature. By expanding the search to include grey literature and hand-searching references within original articles, reviews, and conference proceedings, the risk of selection bias is minimised, and potentially relevant studies are identified. Furthermore, removing the restriction on the year of publication and language allows for a more inclusive approach to capturing relevant literature on the topic.
2. PICO

The revised PICO framework provides a clearer delineation of the participants, interventions, comparisons, and outcomes for the systematic review and meta-analysis. This refined framework ensures a focused and structured approach to addressing the research question and synthesising evidence from relevant studies.
