# Supplementary material for: Can artificial intelligence-driven cephalometric analysis replace manual tracing? A systematic review and meta-analysis
Source: Eur J Orthod. 2024 Jun 19;46(4):cjae029. doi: 10.1093/ejo/cjae029 (PMC11185929; doi:10.1093/ejo/cjae029)
Supplement: cjae029_AQ16_Supplementary_File_2 [file cjae029_aq16_supplementary_file_2.docx]

**Supplementary File 2.** Studies excluded following eligibility criteria screening with reasons (n=45).

| **No** | **Authors, year** | **Reasons for exclusion** |
| --- | --- | --- |
| 1 | Forsyth & Davis, 1996 | Results not synthesizable |
| 2 | Hutton et al., 2000 | Not AI |
| 3 | S. Li et al., 2002 | Unclear AI training |
| 4 | S. P. Li et al., 2002 | Unclear alogrithm |
| 5 | Rueda & Alcañiz, 2006 | Unclear AI training |
| 6 | El-Fegh et al., 2008 | Unclear AI training |
| 7 | Lavergne & Gasson, 2008 | Irrelevant/not enough landmarks |
| 8 | Gupta et al., 2016 | Results not synthesizable |
| 9 | Ed-Dhahraouy et al., 2018 | Unclear AI training |
| 10 | Kang et al., 2019 | Irrelevant/not enough landmarks |
| 11 | Nishimoto et al., 2019 | Results not synthesizable |
| 12 | Porto et al., 2019 | Not using lateral cephalograms |
| 13 | Kunz et al., 2020 | Results not synthesizable |
| 14 | Lachinov et al., 2020 | Irrelevant/not enough landmarks |
| 15 | Meriç & Naoumova, 2020 | Unclear AI training |
| 16 | Yu et al., 2020 | Irrelevant/not enough landmarks |
| 17 | Baksi et al., 2021 | Irrelevant/not enough landmarks |
| 18 | Bermejo et al., 2021 | Irrelevant/not enough landmarks |
| 19 | Chen et al., 2021 | Irrelevant/not enough landmarks |
| 20 | Kang et al., 2021 | Irrelevant/not enough landmarks |
| 21 | Kim et al., 2021 | Not using lateral cephalograms |
| 22 | Kolsanov et al., 2021 | Results not synthesizable |
| 23 | Ahn et al., 2022 | Irrelevant/not enough landmarks |
| 24 | Ghowsi et al., 2022 | Unclear algorithm |
| 25 | Katyal & Balakrishnan, 2022 | Unclear AI training |
| 26 | Ristau et al., 2022 | Unclear AI training |
| 27 | Suhail et al., 2022 | Results not synthesizable |
| 28 | Torres et al., 2022 | Irrelevant/not enough landmarks |
| 29 | Tsolakis et al., 2022 | Unclear AI training |
| 30 | Yassir et al., 2022 | Unclear AI training |
| 31 | Blum et al., 2023 | Irrelevant/not enough landmarks |
| 32 | Cao et al., 2023 | Review article |
| 33 | Duran et al., 2023 | Results not synthesizable |
| 34 | El-Dawlatly et al., 2023 | Results not synthesizable |
| 35 | Gomez-Trenado et al., 2023 | Not using lateral cephalograms |
| 36 | Guinot-Barona et al., 2023 | Results not synthesizable |
| 37 | Indermun et al., 2023 | Unclear AI training |
| 38 | Kunz et al., 2023 | Results not synthesizable |
| 39 | H. Lee et al., 2023 | Not using lateral cephalograms |
| 40 | J. Lee et al., 2023 | Results not synthesizable |
| 41 | Menezes et al., 2023 | Results not synthesizable |
| 42 | Prince et al., 2023 | Unclear AI training |
| 43 | Takahashi et al., 2023 | Not using lateral cephalograms |
| 44 | Ye et al., 2023 | Unclear AI training |
| 45 | Zhou et al., 2023 | Results not synthesizable |

**References**

1. Forsyth DB, Davis DN. Assessment of an automated cephalometric analysis system. *European Journal of Orthodontics* 1996;18(5):471-8.
2. Hutton TJ, Cunningham S, Hammond P. An evaluation of active shape models for the automatic identification of cephalometric landmarks. *European Journal of Orthodontics* 2000;22(5):499-508.
3. Li S, Wu Q, Zhang H. Development of a computerized automatic identification system for use in cephalometry. *Zhonghua kou qiang yi xue za zhi* 2002;37(6):466-8.
4. Li SP, Wu QL, Zhang HY. Automated recognition and identification of soft tissue landmarks in cephalematric analysis. *Zhejiang Da Xue Xue Bao Yi Xue Ban* 2002;31(4):288-90
5. Rueda S, Alcañiz M. An approach for the automatic cephalometric landmark detection using mathematical morphology and active appearance models. *MICCAI* 2006, 12006.159-66.
6. El-Fegh I, Galhood M, Sid-Ahmed M, Ahmadi M. Automated 2-D cephalometric analysis of X-ray by image registration approach based on least square approximator. *IEEE Engineering in Medicine and Biology Society*. 2008;2008:3949-52.
7. Lavergne J, Gasson N. Class II malocclusions studied by neural networks. *L' Orthodontie française.* 2008;79(2):91-7.
8. Gupta A, Kharbanda OP, Sardana V, Balachandran R, Sardana HK. Accuracy of 3D cephalometric measurements based on an automatic knowledge-based landmark detection algorithm. *International Journal of Computer Assisted Radiology and Surgery*. 2016;11(7):1297-309.
9. Ed-Dhahraouy M, Riri H, Ezzahmouly M, Bourzgui F, El Moutaoukkil A. A new methodology for automatic detection of reference points in 3D cephalometry: A pilot study. *International orthodontics*. 2018;16(2):328-37.
10. Kang SH, Jeon K, Kim HJ, Seo JK, Lee SH. Automatic Three-Dimensional Cephalometric Annotation System Using Three-Dimensional Convolutional Neural Networks. *International Journal of Computer Assisted Radiology and Surgery* 2019;14:189-90.
11. Nishimoto S, Sotsuka Y, Kawai K, Ishise H, Kakibuchi M. Personal computer-based cephalometric landmark detection with deep learning, using Cephalograms on the Internet. *The Journal of craniofacial surgery*. 2019;30(1):91-5.
12. Porto LF, Lima LNC, Flores MRP, et al. Automatic cephalometric landmarks detection on frontal faces: An approach based on supervised learning techniques. *Digital Investigation* 2019;30:108-16.
13. Kunz F, Stellzig-Eisenhauer A, Zeman F, Boldt J. Artificial intelligence in orthodontics Evaluation of a fully automated cephalometric analysis using a customized convolutional neural network. *Journal Of Orofacial Orthopedics-Fortschritte Der Kieferorthopadie*. 2020;81(1):52-68.
14. Lachinov D, Getmanskaya A, Turlapov V. Cephalometric Landmark Regression with Convolutional Neural Networks on 3D Computed Tomography Data. *Pattern Recognition and Image Analysis.* 2020;30(3):512-22.
15. Meriç P, Naoumova J. Web-based Fully Automated Cephalometric Analysis: Comparisons between App-aided, Computerized, and Manual Tracings. *Turkish Journal of Orthodontics* 2020;33(3):142-9.
16. Yu HJ, Cho SR, Kim MJ, Kim WH, Kim JW, Choi J. Automated Skeletal Classification with Lateral Cephalometry Based on Artificial Intelligence. *Journal of Dental Research*. 2020;99(3):249-56.
17. Baksi S, Freezer S, Matsumoto T, Dreyer C. Accuracy of an automated method of 3D soft tissue landmark detection. *European Journal of Orthodontics*. 2021;43(6):622-30.
18. Bermejo E, Taniguchi K, Ogawa Y, et al. Automatic landmark annotation in 3D surface scans of skulls: Methodological proposal and reliability study. *Computer Methods and Programs In Biomedicine*. 2021;210.
19. Chen X, Lian C, Deng HH, et al. Fast and Accurate Craniomaxillofacial Landmark Detection via 3D Faster R-CNN. *IEEE Transactions on Medical Imaging* 2021;40(12):3867-78.
20. Kang SH, Jeon K, Kang SH, Lee SH. 3D cephalometric landmark detection by multiple stage deep reinforcement learning. *Scientific Reports* 2021;11(1):17509.
21. Kim MJ, Liu Y, Oh SH, Ahn HW, Kim SH, Nelson G. Evaluation of a multi-stage convolutional neural network-based fully automated landmark identification system using cone-beam computed tomography-synthesized posteroanterior cephalometric images. *Korean Journal of Orthodontics*. 2021;51(2):77-85.
22. Kolsanov AV, Popov NV, Ayupova IO, Tsitsiashvili AM, Gaidel AV, Dobratulin KS. Cephalometric analysis of lateral skull X-ray images using soft computing components in the search for key points. *Stomatologiia* *(Mosk).* 2021;100(4):63-7.
23. Ahn J, Nguyen TP, Kim YJ, Kim T, Yoon J. Automated analysis of three-dimensional CBCT images taken in natural head position that combines facial profile processing and multiple deep-learning models. *Computer Methods and Programs in Biomedicine*. 2022;226.
24. Ghowsi A, Hatcher D, Suh H, et al. Automated landmark identification on cone-beam computed tomography: Accuracy and reliability. *Angle Orthodontist*. 2022;92(5):642-54.
25. Katyal D, Balakrishnan N. Evaluation of the accuracy and reliability of WebCeph - An artificial intelligence-based online software. *APOS Trends in Orthodontics*. 2022;12(4):271-6.
26. Ristau B, Coreil M, Chapple A, Armbruster P, Ballard R. Comparison of AudaxCeph®'s fully automated cephalometric tracing technology to a semi-automated approach by human examiners. *International Orthodontics.* 2022;20(4):100691.
27. Suhail S, Harris K, Sinha G, et al. Learning Cephalometric Landmarks for Diagnostic Features Using Regression Trees. *Bioengineering-Basel*. 2022;9(11).
28. Torres HR, Morais P, Fritze A, et al. 3D Facial Landmark Localization for cephalometric analysis. Annual International Conference of the IEEE Engineering in Medicine and Biology Society*. IEEE Engineering in Medicine and Biology Society*. 2022;2022:1016-9.
29. Tsolakis IA, Tsolakis AI, Elshebiny T, Matthaios S, Palomo JM. Comparing a fully automated cephalometric tracing method to a manual tracing method for orthodontic diagnosis. *Journal of Clinical Medicine* 2022;11(22).
30. Yassir YA, Salman AR, Nabbat SA. The accuracy and reliability of WebCeph for cephalometric analysis. *Journal of Taibah University Medical Sciences* 2022;17(1):57-66.
31. Blum FMS, Möhlhenrich SC, Raith S, et al. Evaluation of an artificial intelligence-based algorithm for automated localization of craniofacial landmarks. *Clinical oral investigations* 2023;27(5):2255-65.
32. Cao L, Yan J, Tang B, Zhao T, Hua F, He H. Research progress on the application of deep learning in cephalometric analysis. *Journal of Prevention and Treatment for Stomatological Diseases* 2023;31(1):58-62.
33. Duran GS, Gökmen Ş, Topsakal KG, Görgülü S. Evaluation of the accuracy of fully automatic cephalometric analysis software with artificial intelligence algorithm. *Orthodontics and Craniofacial Research* 2023;26(3):481-90.
34. El-Dawlatly M, Attia KH, Abdelghaffar AY, Mostafa YA, Abd El-Ghafour M. Preciseness of artificial intelligence for lateral cephalometric measurements. *Journal Of Orofacial Orthopedics-Fortschritte Der Kieferorthopadie* 2023.
35. Gomez-Trenado G, Mesejo P, Cordon O. Cascade of convolutional models for few-shot automatic cephalometric landmarks localization. *Engineering Applications of Artificial Intelligence.* 2023;123.
36. Guinot-Barona C, Alonso Pérez-Barquero J, Galán López L, et al. Cephalometric analysis performance discrepancy between orthodontists and an artificial intelligence model using lateral cephalometric radiographs. *Journal of Esthetic and Restorative Dentistry*. 2023.
37. Indermun S, Shaik S, Nyirenda C, Johannes K, Mulder R. Human examination and artificial intelligence in cephalometric landmark detection-is AI ready to take over? *Dento maxillo facial radiology*. 2023;52(6):20220362.
38. Kunz F, Stellzig-Eisenhauer A, Widmaier LM, Zeman F, Boldt J. Assessment of the quality of different commercial providers using artificial intelligence for automated cephalometric analysis compared to human orthodontic experts. *Journal Of Orofacial Orthopedics-Fortschritte Der Kieferorthopadie* 2023.
39. Lee H, Cho JM, Ryu S, et al. Automatic identification of posteroanterior cephalometric landmarks using a novel deep learning algorithm: a comparative study with human experts. *Scientific Reports* 2023;13(1):15506.
40. Lee J, Bae SR, Noh HK. Commercial artificial intelligence lateral cephalometric analysis: part 2-effects of human examiners on artificial intelligence performance, a pilot study. *The Journal of Clinical Pediatric Dentistry*. 2023;47(6):130-41.
41. Menezes LD, Silva TP, dos Santos MAL, et al. Assessment of landmark detection in cephalometric radiographs with different conditions of brightness and contrast using the an artificial intelligence software. *Dentomaxillofacial Radiology*. 2023;52(8).
42. Prince STT, Srinivasan D, Duraisamy S, Kannan R, Rajaram K. Reproducibility of linear and angular cephalometric measurements obtained by an artificial-intelligence assisted software (WebCeph) in comparison with digital software (AutoCEPH) and manual tracing method. *Dental Press Journal of Orthodontics*. 2023;28(1).
43. Takahashi K, Shimamura Y, Tachiki C, Nishii Y, Hagiwara M. Cephalometric landmark detection without X-rays combining coordinate regression and heatmap regression. *Scientific Reports*. 2023;13(1):20011.
44. Ye H, Cheng Z, Ungvijanpunya N, Chen W, Cao L, Gou Y. Is automatic cephalometric software using artificial intelligence better than orthodontist experts in landmark identification? *BMC Oral Health*. 2023;23(1):467.
45. Zhou Y, Mao B, Zhang J, Zhou Y, Li J, Rong Q. Orthodontic craniofacial pattern diagnosis: cephalometric geometry and machine learning. *Medical and Biological Engineering and Computing*. 2023;61(12):3345-61.
